# Candidate genomic regions underlying capsule shattering in sesame revealed by multi-model GWAS and field-based phenotyping

**DOI:** 10.1007/s00122-026-05151-7

**Published:** 2026-01-20

**Authors:** Mohammed Elsafy, Wafa Badawi, Ahmed Ibrahim, Elamin Hafiz Baillo, A. H. Abu Assar, Haftom Brhane, Umer Mahmood, Prabin Bajgain, Tilal Abdelhalim, Mahbubjon Rahmatov

**Affiliations:** 1 Department of Plant Breeding, Swedish University of Agricultural Sciences (SLU), PO Box 190, 234 22 Alnarp, Sweden; 2 Agricultural Research Corporation (ARC), B.O. Box 126, Wad Medani, Sudan; 3 United States Dairy Forage Research Center, USDA-ARS, 1925 Linden Dr, Madison, WI 53706 USA; 4 Biotechnology and Biosafety Research Center, Agricultural Research Corporation, Shambat, Khartoum North, Sudan

## Abstract

Capsule shattering in sesame is a major agronomic constraint that reduces yield stability and limits mechanized harvesting efficiency. To address this challenge, 200 genetically diverse sesame genotypes from Sudan were genotyped using genotyping-by-sequencing (GBS) and evaluated for three consecutive seasons under field conditions for shattering type (ST), type of capsule beak (TCB), and bicarpellate capsule shape (BS). The resulting phenotypic and genotypic data were integrated into a multi-model genome-wide association study (GWAS) framework (BLINK, FarmCPU, and MLMM) to elucidate the genetic architecture of capsule-shattering traits. Two marker-trait associations (MTAs) were consistently identified across the GWAS models, comprising *Chr1_19419575* associated with the TCB and *Chr2_15649330* linked to ST. Additional MTAs, including *Chr8_31466064* for ST and *Chr8_19392181* and *Chr8_30292484* for TCB, were also detected in this study, further highlighting the complex genetic regulation of capsule traits. Allelic effect analysis further validated the functional role of key allelic variants at *Chr2_15649330* and *Chr8_31466064*, demonstrating significant differences in shattering responses among genotypic subgroups. In silico functional enrichment analysis using a candidate gene approach identified 68 homologous genes associated with pod shattering in *Brassica napus*, of which *FLZ3*, *RZF1*, *MKK5*, and *COR27* showed distinct expression patterns that correlated with shattering susceptibility during pod development. These results provide new insights into the genetic regulation of capsule shattering, providing valuable targets for marker-assisted selection and development of sesame cultivars with enhanced resistance to shattering.

**Supplementary Information:**

The online version contains supplementary material available at 10.1007/s00122-026-05151-7.

## Introduction

Sesame *(Sesamum indicum* L.) is one of the earliest domesticated oilseed crops with a cultivation history spanning several millennia (Najeeb et al. 2012). It is extensively cultivated in tropical and subtropical regions, particularly Asia and Africa, owing to its excellent adaptability to a wide range of agro-climatic conditions (Mehmood et al. 2021). Sesame seeds are highly valued for their rich nutritional composition, which includes high-quality oils, proteins, natural antioxidants, and bioactive lignans, such as sesamin (Elsafy 2023). Oil extracted from sesame seeds is widely recognized for its distinctive flavor, oxidative stability, and numerous health benefits, which contribute to its growing demand in both domestic and international edible oil markets (Mostashari and Mousavi Khaneghah 2024). Despite its agronomic and economic importance, sesame remains vulnerable to several abiotic stresses, specifically drought, salinity, and waterlogging, particularly during germination and early vegetative stages, which can significantly reduce both the yield and seed quality (Dossa et al. 2016; Wang et al. 2018; You et al. 2018). In addition, sesame is affected by a range of biotic stresses, especially fungal diseases, which further reduce productivity and limit its stable cultivation in vulnerable agro-ecological zones (Chowdhury et al. 2017; You et al. 2019).

Although sesame has a long history of domestication, it has not been systematically selected for shattering resistance, which has resulted in most commercial cultivars relying on natural seed dispersal and requiring manual harvesting (Teboul et al. 2022). Sesame fruit capsules split open along the suture lines, releasing seeds onto the ground, which leads to substantial yield loss and prevents mechanized harvesting, thereby restricting large-scale cultivation (Ansah et al. 2015). Anatomical studies in sesame and other crops have demonstrated that capsule shattering is driven by the formation of an abscission layer and the activity of cell wall-degrading enzymes, such as cellulase and polygalacturonase (PG), which weaken structural integrity and promote capsule opening (Yadav et al. 2022). Therefore, breeding approaches that aim to reduce cellulase and PG activities can provide a viable strategy for developing non-shattering varieties. The key anatomical traits influencing capsule shattering in sesame include capsule constriction, membrane integrity, capsule-opening behavior, placenta attachment, and seed positioning. The first naturally occurring mutant with complete shattering resistance, controlled by a single gene pair, was identified in 1946 (Langham 2001). However, this mutant is unsuitable for commercial production because of the difficulty in breaking open the indehiscent capsules and the increased risk of seed damage during processing (Zhang et al. 2018). Subsequent breeding programs have developed improved cultivars that display partial shattering resistance, characterized by capsule opening restricted to the tip, prolonged retention on the plant after maturation, and reduced seed moisture content traits that together enable more efficient harvesting and postharvest handling (Couch et al. 2017).

In sesame, a major quantitative trait locus (QTL) known as *SiCD* has been associated with the indehiscent capsule phenotype, accounting for approximately 76.8% of the phenotypic variation, and representing a key genomic region for breeding non-shattering varieties (Teboul et al. 2022). A genome-wide association study (GWAS) identified *S8_5062843* at 78.9 centimorgans near the distal end of linkage group 8 (chromosome 8), which was strongly associated with capsule-shattering resistance (Yol et al. 2021). The 5′ untranslated region of *SiNST1*, which encodes a transcription factor with an *NAM*, *ATAF1/2*, and *CUC2* (NAC) domain, contains a 1,049-base deletion that may influence capsule dehiscence in sesame by regulating molecular mechanisms (Ju et al. 2024). Regardless of these advances, capsule shattering remains a complex trait governed by interconnected regulatory network of transcription factors and genes involved in cell wall biosynthesis, modification, and remodeling. According to Girin et al. (2010), the transcription factors (INDEHISCENT) is essential for defining the valve margin cell fate and regulating the formation of the dehiscence zone in Arabidopsis, which may serve as a model for understanding similar processes in sesame. These findings demonstrate both the progress made and the continuing need to identify additional loci and candidate genes through advanced approaches, such as multi-model GWAS across multiple environments.

Recent advances in GWAS have demonstrated the importance of integrating multi-environmental phenotyping to identify robust and reproducible marker-trait associations (MTAs), particularly for complex traits influenced by G × E interactions (López-Hernández et al. 2023). Stable QTLs for seed size in soybeans have been consistently detected across multiple seasons (Bhat et al. 2025; López-Hernández et al. 2023), whereas yield-related loci in winter oilseed rape have been validated under contrasting climatic conditions (Corlouer et al. 2024). Similarly, environment-responsive loci for drought and heat tolerance have been identified in common bean × tepary bean populations across diverse locations (López-Hernández et al. 2023), while multi-season studies in safflower have revealed both stable and environment-specific MTAs for yield and oil content (Zhao et al. 2022). As a result, multi-model GWAS frameworks exploit the strengths of different algorithms to enhance detection power and reduce false positives. For instance, in the macauba palm, a combination of BLINK and FarmCPU identified 92 significant SNPs and 12 candidate genes associated with oil-related traits and stress responses (Couto et al. 2024). Comparable strategies in oilseed rape and other crops have proven effective in dissecting complex traits, indicating the relevance of applying multi-environment and multi-model GWAS to capsule shattering in sesame, a trait characterized by genetic complexity and strong environmental sensitivity, which has long hindered breeding progress (Raman et al. 2014).

The present study aimed to identify genomic regions and candidate genes associated with capsule shattering through a multi-model GWAS approach, using a diverse panel of sesame genotypes from Sudan, a center of genetic diversity for the crop. Investigating the genetic architecture of shattering in these locally adapted genotypes will enable the discovery of allelic variations and the development of molecular tools to support the marker-assisted selection. These findings are expected to accelerate the breeding of shattering-resistant sesame cultivars, thereby enhancing yield stability, facilitating mechanized harvesting, and contributing to the long-term sustainability of sesame production on both national and global scales.

## Materials and methods

### Plant materials

This study utilized a panel of 200 diverse sesame genotypes, comprising genebank accessions from the Agricultural Plant Genetic Resources Conservation and Research Center, Sudan (APGRC), landraces, advanced breeding lines, and released varieties.

### Experiment setup

The field trial was conducted over three consecutive growing seasons (2021, 2022, and 2023) at the Matuq Research Station in Gezira State, Sudan (14° 11′ 10″ N, 32° 34′ 48′ E). This region has a mean annual high temperature of 37°C and a mean low temperature of 26 °C. The climate is characterized by an average annual precipitation of 26 mm, an average relative humidity of 28%, and approximately 41 rainy days per year, with each rainfall event contributing at least 1.0 mm.

An augmented block design was employed to evaluate 200 sesame genotypes together with three control checks: Gadarif, Kenana 2, and Promo. The experimental layout consisted of eight blocks, each containing 28 plots of 4 m^2^. Within each block, 25 unique genotypes were randomly selected, while the checks were replicated across all blocks to allow for inter-block variation. In this augmented design, the tested genotypes were not replicated within blocks, whereas the three control varieties were replicated in each block to enable adjustment for spatial variation. The following measures were employed to minimize the experimental error: Field heterogeneity was managed by dividing the trial into eight smaller blocks with randomized plots, and uniform agronomic practices and border rows were applied to reduce edge effects. Spatial analyses, including nearest-neighbor and AR1 × AR1 models, were used to generate control-adjusted means, thereby improving accuracy despite the lack of replication of the test genotypes. Furthermore, multi-season evaluations across three years, combined with mixed-model analysis, enabled the partitioning of genetic, spatial, and temporal variance, which further enhanced the precision and reliability of trait estimates.

### Phenotyping for shattering

Phenotypic evaluations were conducted under field conditions during all seasons (2021, 2022, and 2023). Three morphological capsule-related traits were assessed across all genotypes: bicarpellate capsule shape (BS), type of capsule beak (TCB), and shattering type (ST). For each accession, mature capsules from five randomly selected plants out of 15 were manually scored using standardized descriptors adapted from the sesame characterization guidelines (IBPGR 2004) (Table 1). To reduce bias, the same trained individual performed all evaluations across all seasons, and uniform scoring procedures were followed. The trait ST was classified into categorical classes (non-shattering, partially shattering, completely shattering) following established same-descriptor sets, while BS and TCB were categorized according to defined morphological classifications (Table 1).

**Table 1:** Qualitative traits and their categorical descriptions in sesame capsule morphology and shattering behavior

Traits	Description
Bicarpellate capsule shape (BS)	(1) Tapered at apex
	(2) Narrow oblong
	(3) Broad oblong
	(4) Square
Type of capsule beak (TCB)	(1) Short
	(2) Long
	(3) Curved
	(4) Cleft
	(5) Other
Shattering type (ST)	(1) Non-shattering
	(2) Partially shattering
	(3) Completely shattering

### DNA isolation, genotyping-by-sequencing, and SNP calling

Young leaf tissues (~ 5 mm in diameter) were collected from each plant and placed in a 96-well plate designed for tissue collection. Genomic DNA was extracted using the Qiagen BioSprint 96 system combined with the Qiagen BioSprint DNA Plant Kit. DNA concentrations were normalized and sequencing libraries were prepared following the genotyping-by-sequencing (GBS) protocol described by Poland et al. (2012). The restriction enzymes, *PstI* and *MspI*, were used to generate genome-wide cuts. The resulting fragments were ligated with unique barcode adapters, multiplexed (96 samples per lane), and sequenced on a NovaSeq 6000 platform (Illumina) at the University of Minnesota Genomics Center (St. Paul, MN, USA).

GBS generated a total of 487.5 million raw reads (mean ± SD per sample = 2.44 ± 0.39 million), with an average Q30 score of 92.8%. After demultiplexing and adapter trimming, 84.3% of reads were uniquely mapped to the *Sesamum indicum* v3.0 reference genome (Wang et al. 2022) using the Burrows–Wheeler aligner (BWA) v0.7.4 (Li and Durbin 2009). SNP calling was performed using SAMtools and BCFtools (Li, 2011), yielding 61,611 raw SNPs. Variants were filtered using a minor allele frequency (MAF) threshold of ≥ 0.03 and a missing data rate of ≤ 20%, yielding a final dataset of 3,636 high-quality SNPs distributed across 13 chromosomes and 17 high-confidence scaffolds. The chosen MAF threshold ensured adequate allele representation in the panel (n = 200) while minimizing the inclusion of low-frequency variants with limited statistical power.

### Genomic data analysis and population structure assessment

To explore genetic relatedness among the genotypes, TASSEL version 5.2.60 (Bradbury et al. 2007) was employed to calculate a similarity matrix using the identity-by-state (IBS) algorithm with 10,000 bootstrap iterations. The resulting matrix was displayed as a heatmap using the R package “heatmap” (Kolde and Kolde 2015). Principal component analysis (PCA) was conducted on the genotypic data to investigate the population structure of the genotypes using the *prcomp* function of the base *stats* package in R, and a scatter plot was generated using *ggplot2* (Wickham and Wickham 2016).

LD decay was evaluated by calculating pairwise LD among markers in TASSEL 5 using a sliding window of 50 markers. The resulting *r*^*2*^ values were plotted against physical distances according to the sesame reference genome v3.0, and a locally weighted scatterplot smoothing (LOWESS) function was applied to illustrate the decay trend. Following Hill and Weir (1988), the LD decay distance was estimated based on the physical distance at which *r*^*2*^ declined below the chosen threshold, and the physical span of the SNP-based LD heatmap was used to visualize genome-wide LD patterns.

### Phenotypic data analysis

Phenotypic data for three capsule-related traits, BS, TCB, and ST, were analyzed using R (R Core Team, 2023). Initial trait distributions were explored using bar plots and frequency figures, and Pearson’s Chi-square tests were used to evaluate significant differences in categorical trait levels across seasons (Duke et al. 2020). Ordinal trait scores were subjected to PCA using the *prcomp* function to identify the principal axes of phenotypic variation. Multiple correspondence analysis (MCA) was conducted using the *FactoMineR* (Lê et al. 2008) and *factoextra* (Kassambara and Mundt 2017) packages to investigate the relationships among trait categories. G × E was visualized by plotting mean trait scores per genotype across seasons using *ggplot2*, with crossing lines indicating interaction effects. For genetic evaluation, the best linear unbiased estimates (BLUE) were obtained using *ASReml,* treating genotype as a fixed effect and block and genotype × environment as random effects.

$$Y_{ijk} = \mu + G_{i} + E_{j} + \left( {GxE} \right)_{ij} + B_{k} + \in_{ijk}$$

where ***Y***_***ijk***_ is the trait value for genotype *i* in environment *j*, block *k,* **µ** is the overall average trait value across all genotypes and environments, **G**_***i***_ is a fixed effect of genotype *i*, **E**_***j***_ is a fixed effect of environment, **(GxE)**_***ij***_ is a random G × E interaction, **B**_***k***_ is a random effect of block, and **ϵ**_***ijk***_ is residual error.

The best linear unbiased predictions (BLUPs) were estimated using a model that treated the genotype as a random effect.

$$Y_{ik} = \mu + G_{i} + B_{k} + \in_{ijk}$$

where **G**_***i***_  ~ N (0, σ^2^_G_) is the random genetic effect, **B**_***k***_ ~ N (0, σ^2^_B_) is the random block effect, and **ϵ**_***ik***_ ~ N (0, σ^2^_e_) is the residual error.

Broad-sense heritability was calculated using the Cullis method based on the variance components and the average pairwise prediction error variance (squared Euclidean distance matrix), as recommended by Cullis et al. (2006). Broad-sense heritability was calculated only for ST as an ordinal or continuous variable, while BS and TCB were ignored as nominal categories unsuitable for heritability analysis.

$$H^{2 } = 1 - \frac{{{\mathrm{mean}}\;{\mathrm{PEV}}}}{{2\sigma_{G}^{2} }}$$

where **PEV** is the average prediction error variance from the matrix of squared standard errors of pairwise differences between genotype predictions, **σ**^**2**^_***G***_ is the genetic variance component (from the BLUP model), and the factor of **2** adjusts for the average variance of pairwise genotype differences.

### Genome-wide association analysis (GWAS)

GWAS were performed using genotype BLUEs as phenotypes and the filtered SNP dataset as genotypic markers. Three GWAS models: fixed and random model circulating probability unification (FarmCPU), Bayesian information and linkage-disequilibrium iteratively nested keyway (BLINK), and multiple locus mixed model (MLMM), were implemented in GAPIT3 package (Wang and Zhang 2021). Significant SNPs were determined using a LOD (-log₁₀ P) threshold of 3.1. This corresponds to a *p value* cutoff of approximately 0.00079. Manhattan and Q-Q plots were generated to illustrate the marker-trait associations based on this significance level.

### Allelic effect analysis

From the GWAS results, the two most significant SNPs associated with ST (shattering type) were selected for downstream single-marker allelic effect analysis. For each locus, genotypic classes were defined using the called allele, and the per-genotype BLUEs for ST were used as the response variables. A one-way ANOVA was conducted with the SNP allele as a fixed factor, followed by Tukey’s honest significant difference (HSD) test to compare the mean differences among allele classes. Allelic effect distributions were visualized using violin and box plots, with Tukey group letters superimposed, in R v4.3.2 with the *dplyr* (Wickham et al. 2019)*, psych* (Revelle and Revelle 2015), *MultcompView* (Graves et al. 2015), and *ggplot2* packages.

### Candidate gene analysis

To identify genes potentially associated with capsule shattering, we focused on the TCB and ST genomic regions flanking the significant SNPs consistently detected across all three GWAS methods. Specifically, putative protein-coding genes located within ± 218,791 bp upstream and downstream of each significant SNP were examined using the updated sesame genome assembly and annotation (Wang et al. 2022). This distance was chosen based on the observed genome-wide mean linkage disequilibrium in sesame, which extended up to 218,791 bp. Genes located within that region were identified, and their Arabidopsis homologs were determined using the Basic Local Alignment Search Tool for Proteins (BLASTP) program. The identified genes were searched against the *Arabidopsis thaliana* proteome database (TAIR11) using an E-value cutoff of 1 × 10^ (-5) (Mahmood et al. 2021). Gene ontology (GO) terms and gene functions were assessed using *Arabidopsis thaliana*. Accordingly, we selected the genes associated with (GO) terms related to pod shattering and seed release. The *A. thaliana* genes were used as queries to perform a BLASTP search (E-value cutoff of 1e-5) against the *Brassica napus* protein sequences available in Chao et al. (2020). Subsequently, expression data were obtained from a *B. napus* pod-shattering study that included RNA sequencing of shatter-resistant and susceptible genotypes sampled at six stages of pod development (Mahmood et al. 2023). The corresponding raw sequencing data are publicly available at the BIG Data Center (BIGD) under the BioProject accession number *PRJCA008288*. A heatmap was generated using Log2(FPKM + 1) expression values, followed by Z-score normalization. Visualization was performed using the R package *pheatmap*.

## Results

### Phenotypic variation

**Fig. 1:**
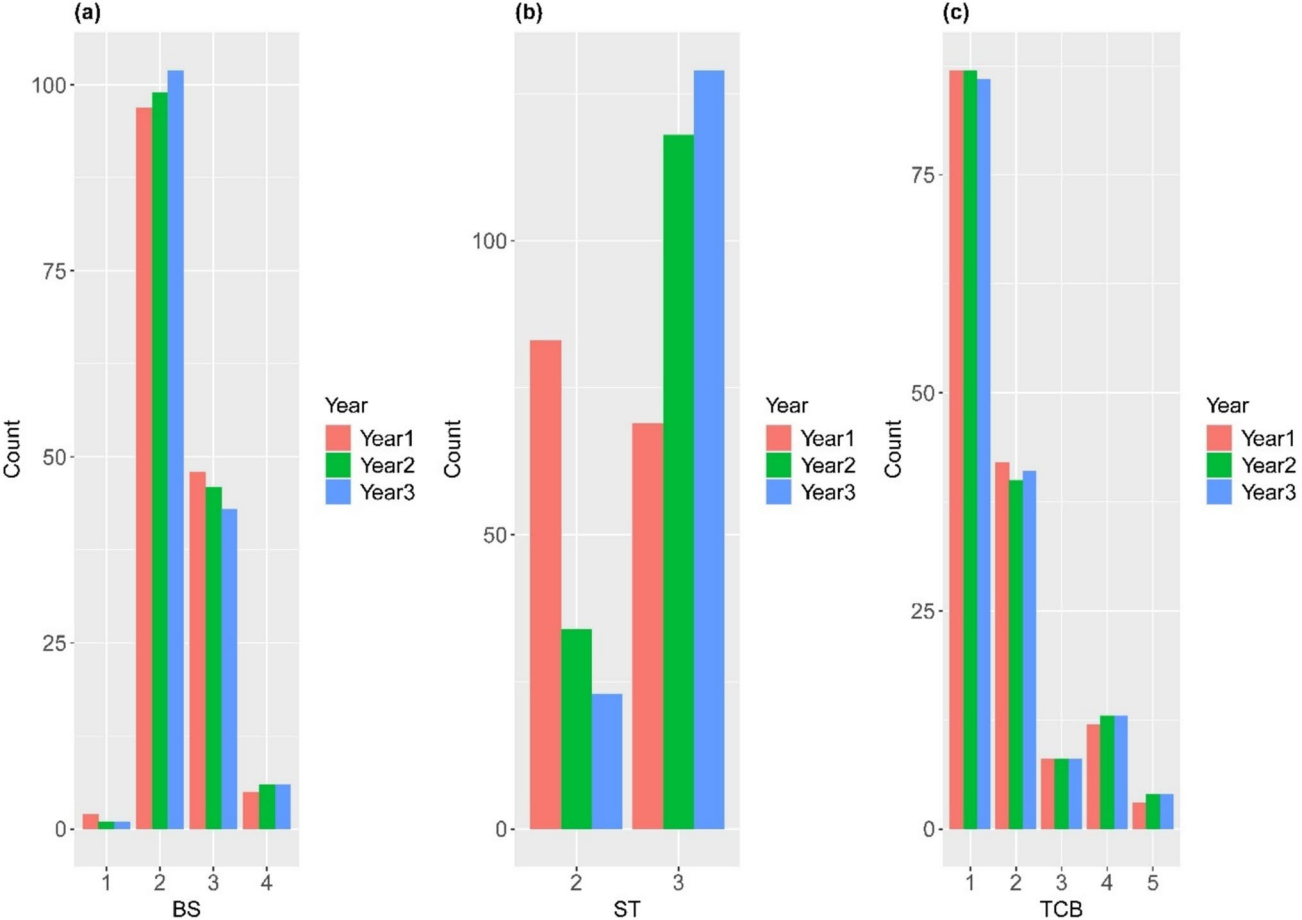
Frequency distribution of the three qualitative traits across the three years in sesame genotypes. **a** Bicarpellate capsule shape (BS), **b** shattering type (ST), and **c** type of capsule beak (TCB)

The frequency distributions of BS, TCB, and ST showed consistent patterns with interannual variation (Fig. 1). The most frequent BS shape was narrow oblong (category 2), observed in 97, 99, and 102 genotypes in years 1, 2, and 3, respectively. Broad oblong (category 3) was the second most frequent genotype, with 48, 46, and 43 genotypes. Tapered at the apex (category 1) and square (category 4) shapes occurred in fewer than seven genotypes each year (Fig. 1a).

A clear temporal shift in the ST categories was observed. In year 1, the partially shattering (category 2; *n* = 83) and completely shattering (category 3; *n* = 69) genotypes were relatively balanced. However, complete shattering increased significantly in years 2 (*n* = 118) and 3 (*n* = 129), whereas partial shattering declined to 34 and 23, respectively. The non-shattering type (Category 1) was absent throughout the study period (Fig. 1b).

For TCB, the short beak type (category 1) was predominant across all years, observed in 87, 87, and 86 genotypes. The long beak type (category 2) was the second most frequent, followed by cleft (category 4), curved (category 3), and other rare and atypical morphologies grouped under category 5, which included asymmetrical beaks, flattened beaks, and irregularly split tips. This category accounted for less than 3% of the genotypes in each year of evaluation (Fig. 1c).

**Fig. 2:**
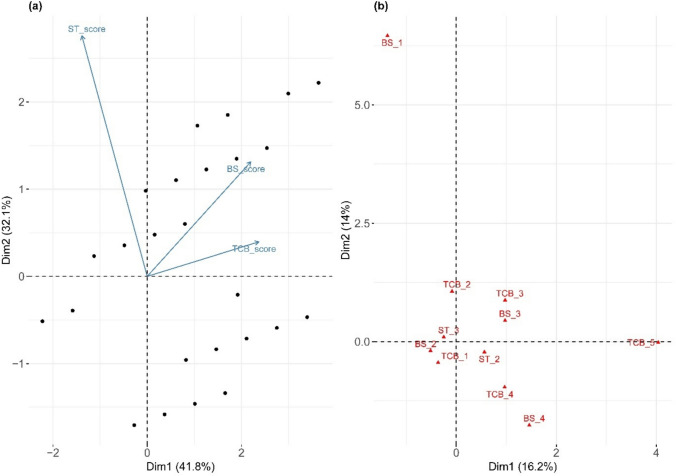
Ordination analysis of three qualitative capsule traits in sesame genotypes. **a** Principal component analysis (PCA) biplot showing the distribution of genotypes based on bicarpellate capsule shape (BS), shattering type (ST), and type of capsule beak (TCB), with trait vectors indicating their contributions to the principal components. (Filled black circle) represents clusters of genotypes with overlapping positions. **b** Multiple correspondence analysis (MCA) plot illustrating the associations between different categorical scores of BS, ST, and TCB

The first principal component (Dim1) accounted for 41.8% of the total variation in BS, TCB, and ST, whereas the second component (Dim 2) accounted for an additional 32.1% (Fig. 2a). Together, these two components captured nearly three-quarters (73.9%) of total variation. ST and TCB showed the highest loadings for Dim1, indicating their dominant role in differentiating genotypes, whereas BS was more strongly associated with Dim2. No apparent clustering by year was detected, suggesting that temporal variation contributed less to overall diversity than inherent trait differences.

For categorical variables, the first dimension (Dim1) of the MCA explained 16.2% of the variation, and the second dimension (Dim2) explained 14%, for a total of 30.2% (Fig. 2b). Similar to PCA, the MCA plot revealed no distinct separation by year but highlighted strong trait-driven associations, particularly between ST and TCB.

The broad-sense heritability (H^2^) for ST was estimated to be 0.39, indicating that approximately 39% of the variation in the shattering type was attributable to genetic differences among the genotypes. BS and TCB did not differ significantly among years (BS: χ^2^ = 1.0, *df* = 6,  *P* = 0.9847; TCB: χ^2^ = 0.3, *df* = 8,  *P* = 1), whereas ST showed a highly significant year effect (χ^2^ = 63.1, *df* = 2, * P* = 1.984 × 10^−14^). Logistic regression analysis confirmed that the odds of complete shattering relative to partial shattering were markedly higher in years 2 and 3 than in year 1 (*P* < 0.001).

**Fig. 3:**
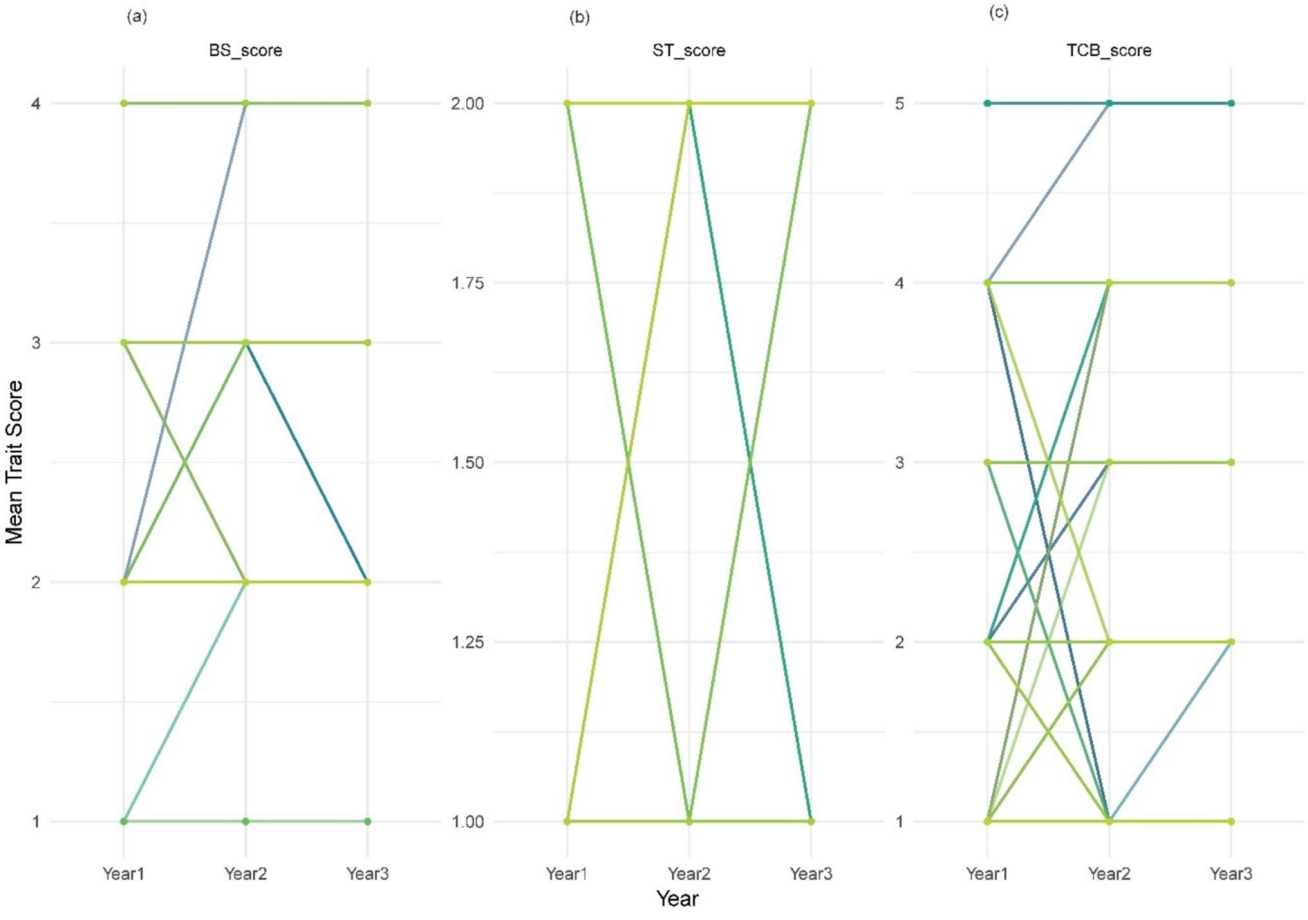
Genotype × environment interaction plots for capsule-related traits in sesame across three environments (Years 1, 2, and 3). Each line represents the mean score per year for each genotype for (**a**) bicarpellate capsule shape (BS), **b** shattering type (ST), and **c** capsule beak type (TCB). Traits were scored as ordinal values and plotted to visualize the genotype stability or responsiveness across environments. Crossing lines indicates genotype × environment interactions

Genotype × environment (G × E) analysis revealed pronounced crossover interactions for ST, indicating strong environmental modulation of shattering responses. Despite the insignificant year effect on TCB, some genotypes exhibited category shifts between years, implying an environmental component at the genotype level. In contrast, BS remained comparatively stable, with most genotypes retaining the same capsule shape classification across the seasons (Fig. 3).

### Population structure, kinship, and linkage disequilibrium analysis

**Fig. 4:**
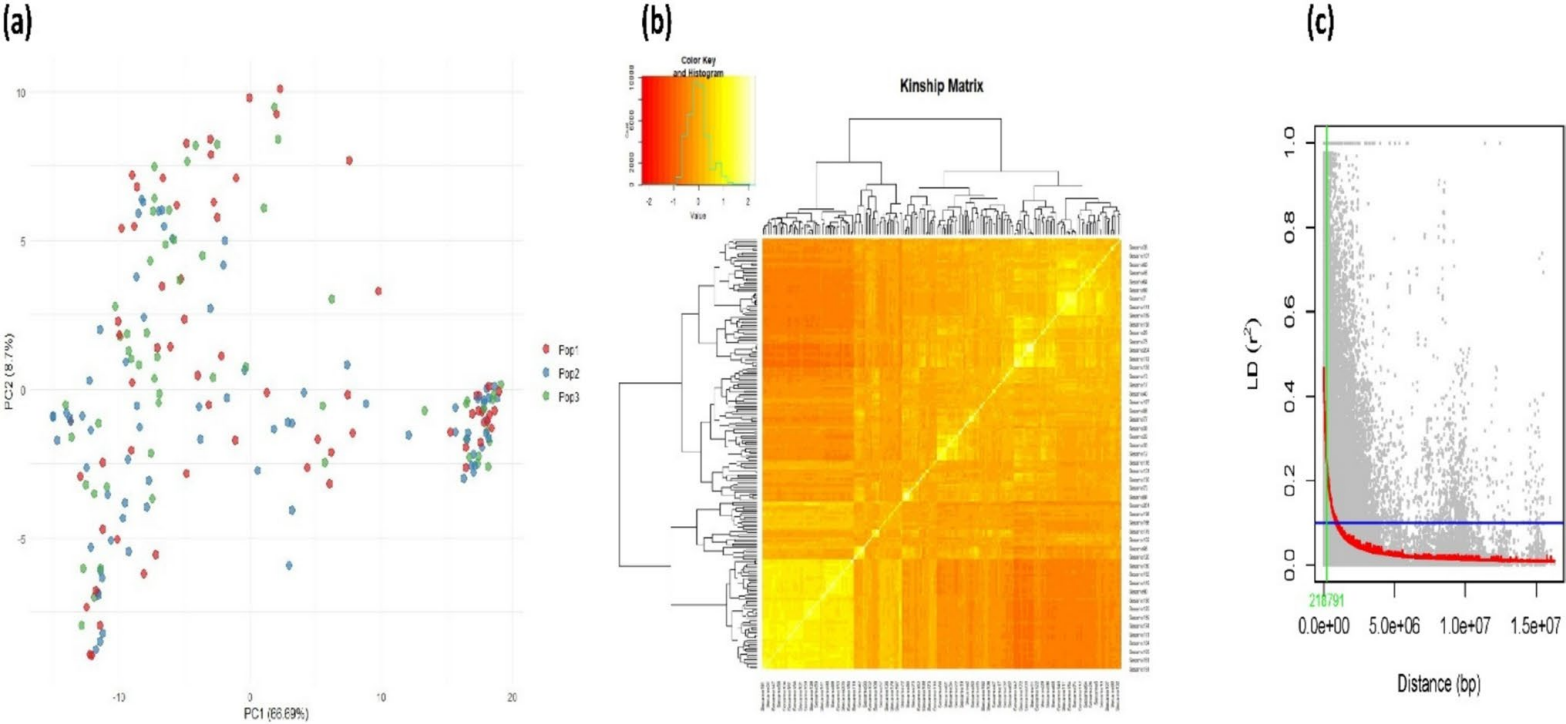
Genetic diversity and population structure analysis in sesame genotypes. **a** Principal component analysis (PCA) plot showing the population structure based on genome-wide SNP data, with individuals grouped into three populations (Pop1, Pop2, and Pop3). **b** Kinship matrix heatmap illustrating genetic relatedness among genotypes, with hierarchical clustering revealing population substructure. **c** Linkage disequilibrium (LD) decay plot showing the relationship between physical distance (in base pairs, bp) and LD (*r*^*2*^), which indicates the extent of LD decay across the genome

Genotypic variation among the 200 sesame genotypes was assessed using PCA, where PC1 accounted for 66.69% of the total genetic variation, and PC2 explained for an additional 8.7% (Fig. 4a). Three population groups (Pop1, Pop2, and Pop3) were identified, but they all showed substantial overlap, indicating partial admixture rather than strict separation. A small subset of genotypes formed a distinct cluster along the positive PC1 axis, reflecting a notable genetic divergence. The Kinship heatmap (Fig. 4b) showed moderate to high relatedness within specific subgroups, consistent with the partial clustering in the PCA. The subclusters in the dendrogram further confirmed the groups of closely related lines with similar characteristics (Fig. 4b). LD decay analysis (Fig. 4c) indicated that *r*^*2*^ dropped below 0.2 at approximately 218 kb, suggesting a relatively rapid decay in this panel. This pattern indicates that high-resolution association mapping in sesame would require a sufficiently dense marker set to capture variations at this scale.

### Identification of significant MTAs related to capsule shattering

**Fig. 5:**
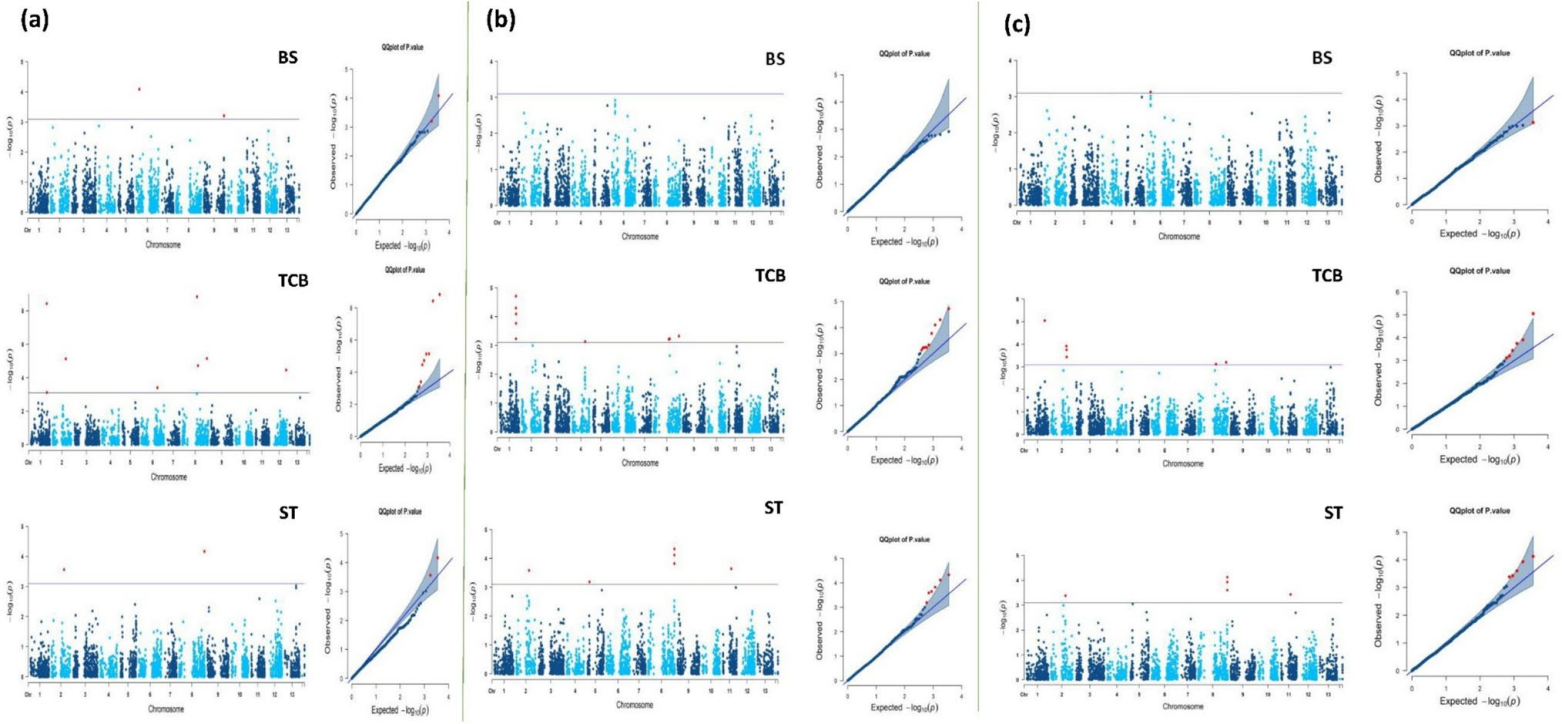
Genome-wide association study (GWAS) results for three qualitative traits in sesame plants using different statistical models. **a** BLINK model, **b** FarmCPU model, and **c** MLMM model. Each panel presents Manhattan plots and corresponding Q-Q plots for the bicarpellate capsule shape (BS), type of capsule beak (TCB), and shattering type (ST). Significant associations were marked above the threshold line, and Q-Q plots were used to assess the fit of observed vs. expected *p* values, indicating the control of false positives and model performance

GWAS identified multiple marker-trait associations (MTAs) for BS, TCB, and ST, with five SNPs consistently detected across all three methods (BLINK, FarmCPU, and MLMM) (Table 2; Fig. 5). For TCB, three reproducible SNPs, *Chr1_19419575*, *Chr8_19392181*, and *Chr8_30292484*, were associated with narrower or more recessed capsule beak morphologies, explaining 13–39% of the phenotypic variance. For ST, two SNPs, *Chr2_15649330* and *Chr8_31466064*, were repeatedly identified across GWAS models. The SNP *Chr8_31466064* was located within a region containing several closely spaced significant markers on chromosome 8, indicating a localized genomic region associated with reduced shattering. These SNPs showed positive allelic effects and moderate coefficients of determination (*R*^2^ up to 0.25), supporting their role in the genetic regulation of capsule dehiscence. Although the individual models detected additional SNPs, five cross-validated SNPs were selected for their statistical robustness, repeated detection, and biological relevance. These genes are strong candidates for functional validation and marker-assisted breeding to enhance shattering resistance in sesame plants.

**Table 2:** Significant SNP markers associated with BS, TCB, and ST traits in sesame were identified using the BLINK, Farm CPU, and MLMM GWAS models

Models	Traits	SNP Marker	Chr	Position	*P *value	MAF	*R*^2^ (%)	Effect
Blink	BS	Chr6_3750656	6	3,750,656	8E−05	0.10	18	0.2
	BS	Chr9_25744188	9	25,744,188	6E−04	0.33	8	− 0.2
	TCB	**Chr1_19419575**	1	19,419,575	4E−09	0.10	21	− 0.8
	TCB	Chr1_19527183	1	19,527,183	8E−04	0.33	3	0.3
	TCB	Chr12_17654820	12	17,654,820	3E−05	0.46	4	0.4
	TCB	Chr2_16485861	2	16,485,861	7E−06	0.46	4	0.3
	TCB	Chr6_19934920	6	19,934,920	4E−04	0.22	2	− 0.2
	TCB	Chr8_18222831	8	18,222,831	1E−09	0.20	7	0.5
	TCB	**Chr8_19392181**	8	19,392,181	2E−05	0.06	31	− 0.6
	TCB	**Chr8_30292484**	8	30,292,484	7E−06	0.42	7	− 0.4
	ST	**Chr2_15649330**	2	15,649,330	3E−04	0.07	25	0.1
	ST	**Chr8_31466064**	8	31,466,064	7E−05	0.35	9	0.1
FarmCPU	TCB	Chr1_19419569	1	19,419,569	5E−05	0.08	0	− 0.8
	TCB	**Chr1_19419575**	1	19,419,575	2E−05	0.10	0	− 0.7
	TCB	Chr1_19459274	1	19,459,274	2E−04	0.02	0	− 1.0
	TCB	Chr1_19526510	1	19,526,510	6E−04	0.35	57	0.4
	TCB	Chr4_15924477	4	15,924,477	7E−04	0.09	0	− 0.5
	TCB	Chr8_18191563	8	18,191,563	6E−04	0.05	1	− 0.7
	TCB	**Chr8_19392181**	8	19,392,181	6E−04	0.06	0	− 0.6
	TCB	**Chr8_30292484**	8	30,292,484	5E−04	0.42	0	− 0.5
	ST	Chr11_10701064	11	10,701,064	2E−04	0.05	15	0.2
	ST	**Chr2_15649330**	2	15,649,330	3E−04	0.07	0	0.2
	ST	Chr5_3576747	5	3,576,747	6E−04	0.21	3	0.1
	ST	Chr8_31466038	8	31,466,038	2E−04	0.37	0	0.1
	ST	Chr8_31466055	8	31,466,055	8E−05	0.36	0	0.1
	ST	**Chr8_31466064**	8	31,466,064	5E−05	0.35	7	0.1
MLMM	BS	Chr6_3750656	6	3,750,656	7E−04	0.10	17	0.2
	TCB	**Chr1_19419575**	1	19,419,575	9E−06	0.10	16	− 0.7
	TCB	Chr2_16485861	2	16,485,861	1E−04	0.46	4	0.4
	TCB	Chr2_16579717	2	16,579,717	2E−04	0.45	0	0.4
	TCB	Chr2_16778004	2	16,778,004	4E−04	0.48	0	0.4
	TCB	**Chr8_19392181**	8	19,392,181	8E−04	0.06	39	− 0.6
	TCB	**Chr8_30292484**	8	30,292,484	6E−04	0.42	7	− 0.5
	ST	Chr11_10701064	11	10,701,064	4E−04	0.05	18	0.2
	ST	**Chr2_15649330**	2	15,649,330	4E−04	0.07	0	0.1
	ST	Chr8_31466038	8	31,466,038	2E−04	0.37	0	0.1
	ST	Chr8_31466055	8	31,466,055	1E−04	0.36	7	0.1
	ST	**Chr8_31466064**	8	31,466,064	8E−05	0.35	14	0.2

*Chr* chromosome, *MAF* minor allele frequency, and *R*^2^ percentage of variance explained by marker

### Allelic effects on significant SNPs

**Fig. 6:**
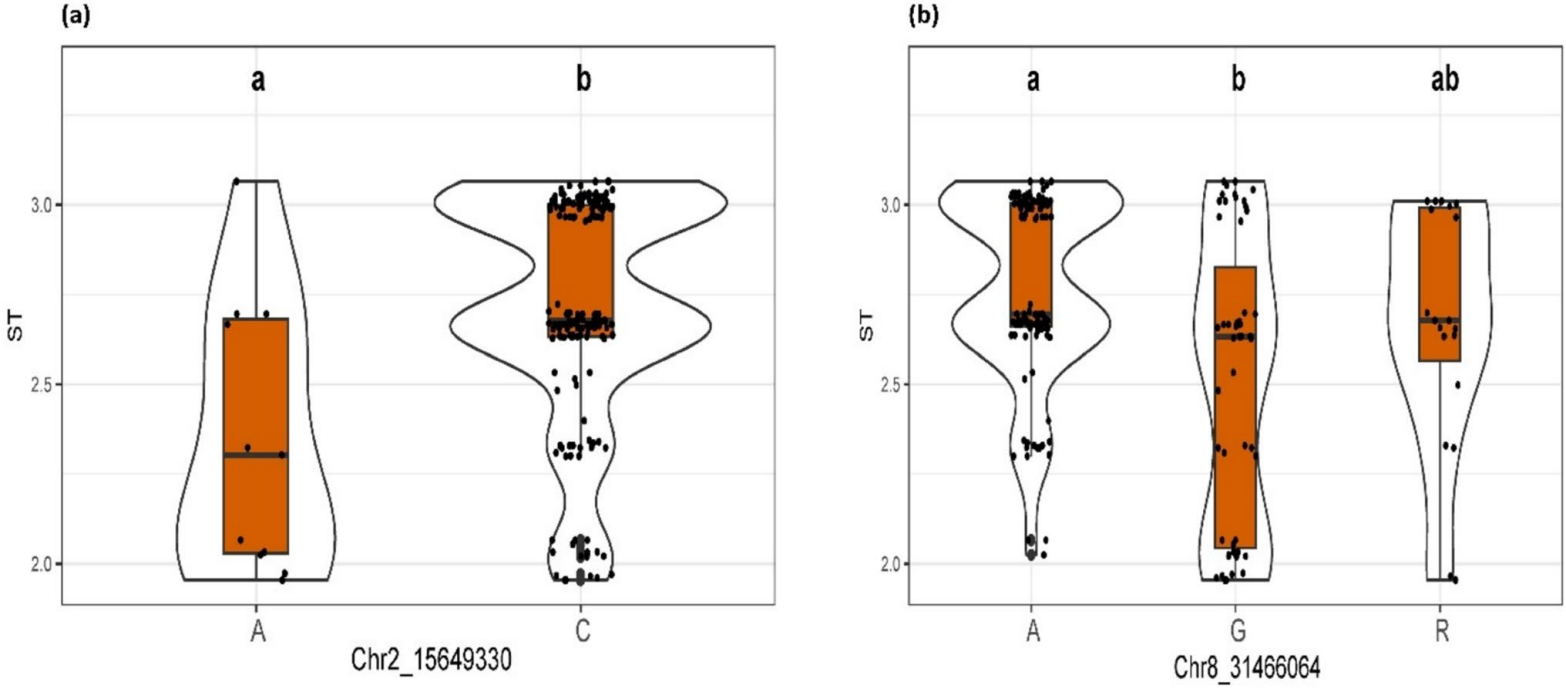
Allelic effects of two significant SNPs associated with shattering type (ST) in sesame. **a** *Chr2_15649330* and **b** *Chr8_31466064*. Violin plots overlaid with box plots show the distribution of ST scores for each allelic class. Letters below the x-axis denote the nucleotide alleles observed at each SNP (A and C for *Chr2_15649330*; A, G, and R for *Chr8_31466064*, where R represents a heterozygous or ambiguous A/G genotype). Black dots represent individual genotypic observations. Letters above the plots indicate statistically homogeneous groups based on Tukey’s HSD test (*P* < 0.01); allelic classes sharing the same letter are not significantly different

At *Chr2_15649330* (Fig. 6a), significant differences in ST were observed among the allele classes. Genotypes carrying the C allele exhibited higher ST values, indicating more complete shattering than those carrying the A allele (Tukey’s HSD, *P* < 0.01). *At Chr8_31466064* (Fig. 6b), the G allele was associated with lower ST values, consistent with reduced shattering, whereas the A allele had the highest median ST. The heterozygous or rare (R) allele class displayed intermediate effects (*P* < 0.01). Violin and boxplot visualizations, which clearly illustrated the distinct distributions between allele classes, consistently supported phenotypic contrasts across allele classes. These results confirmed that *Chr2_15649330* and *Chr8_31466064* had measurable effects on shattering responses, validating their functional importance.

### Candidate genes and functional analysis

**Fig. 7:**
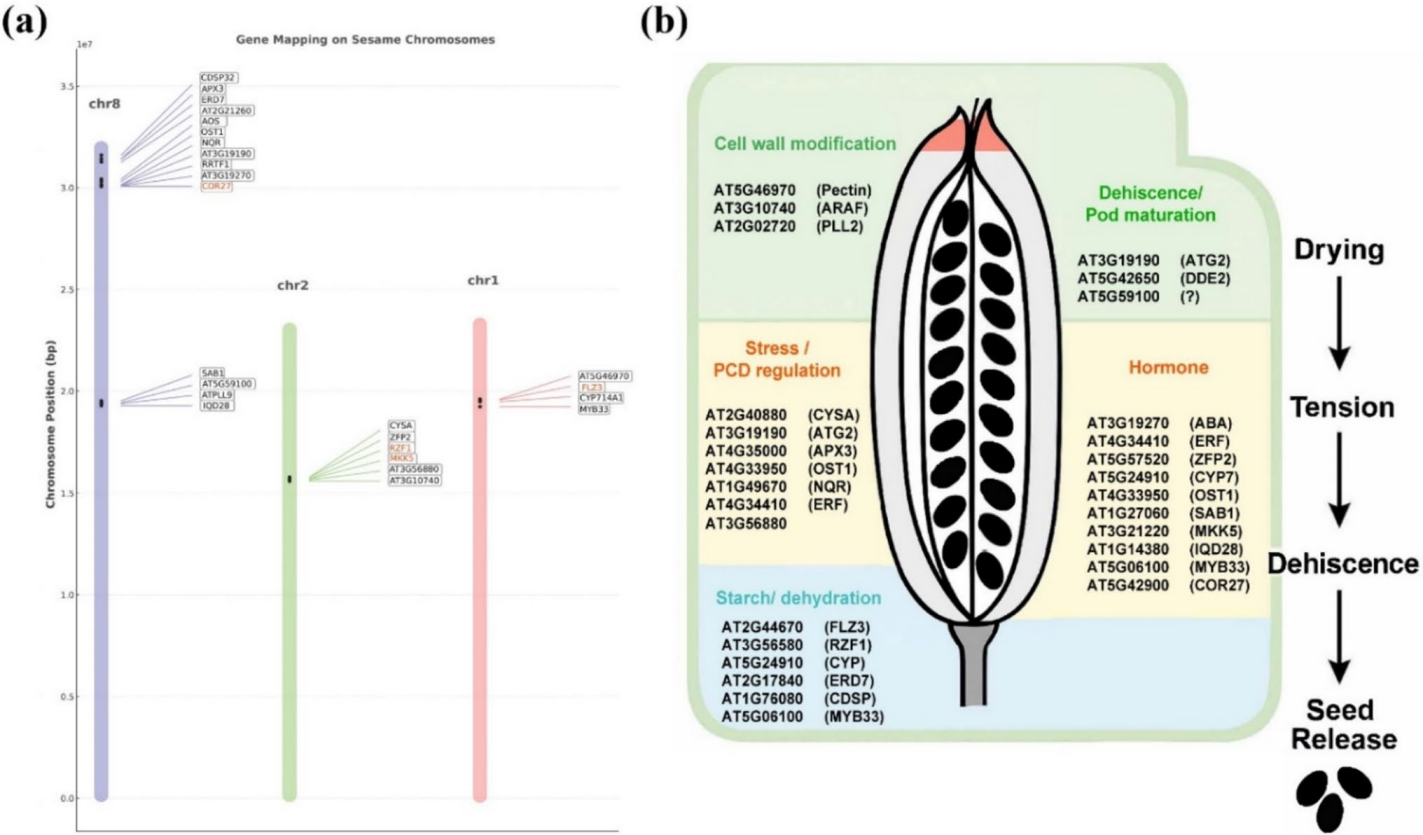
Candidate gene identification and functional categorization related to pod shattering in sesame. **a** Genomic mapping of candidate genes on sesame chromosomes (*Chr1*, *Chr2*, and *Chr8*) surrounding significant SNPs identified through GWAS. Homologous genes were identified based on annotations from *A. thaliana*, focusing on genes related to pod shattering and seed release. **b** Functional classification of the identified candidate genes based on their roles in biological processes related to pod dehiscence. Genes were grouped into five categories: cell wall modification, stress/PCD regulation, starch metabolism/dehydration, hormonal regulation, and dehiscence/pod maturation. The right panel illustrates a conceptual model linking gene function to the physiological stages of pod shattering: drying, tension buildup, dehiscence, and seed release

A total of 273 genes were identified within the genomic regions flanking the significant SNPs, including 25 genes functionally linked to pod shattering and seed release, based on annotations in *A. thaliana* (Fig. 7A). These genes were located within a 437,582 bp window surrounding the significant SNPs detected by the GWAS models (Table S1). BLASTP analysis against the *B. napus* protein database further identified 68 homologous proteins, whose transcriptional profiles during pod development and dehiscence were subsequently examined. These genes spanned functional categories, including cell wall modification (pectin methylesterase inhibitor, *ARAF*, *PLL2*), stress and programmed cell death (PCD) regulation (*CYSA*, *ATG2*, *APX3*, *ERF*, *RZF1*), starch metabolism and dehydration (*FLZ3*, *RZF1*, *CYP*, *ERD7*, *CDSP*), and hormone signaling (*ERF*, *ZFP2*, *CYP*, *OST1*, *MKKS*, *1QD28*, *MYB33*, and *COR27*) (Table S2). Among these, *ATG2*, *DDE2*, and *SAB1* emerged as key players in pod dehiscence and maturation (Fig. 7b).

**Fig. 8:**
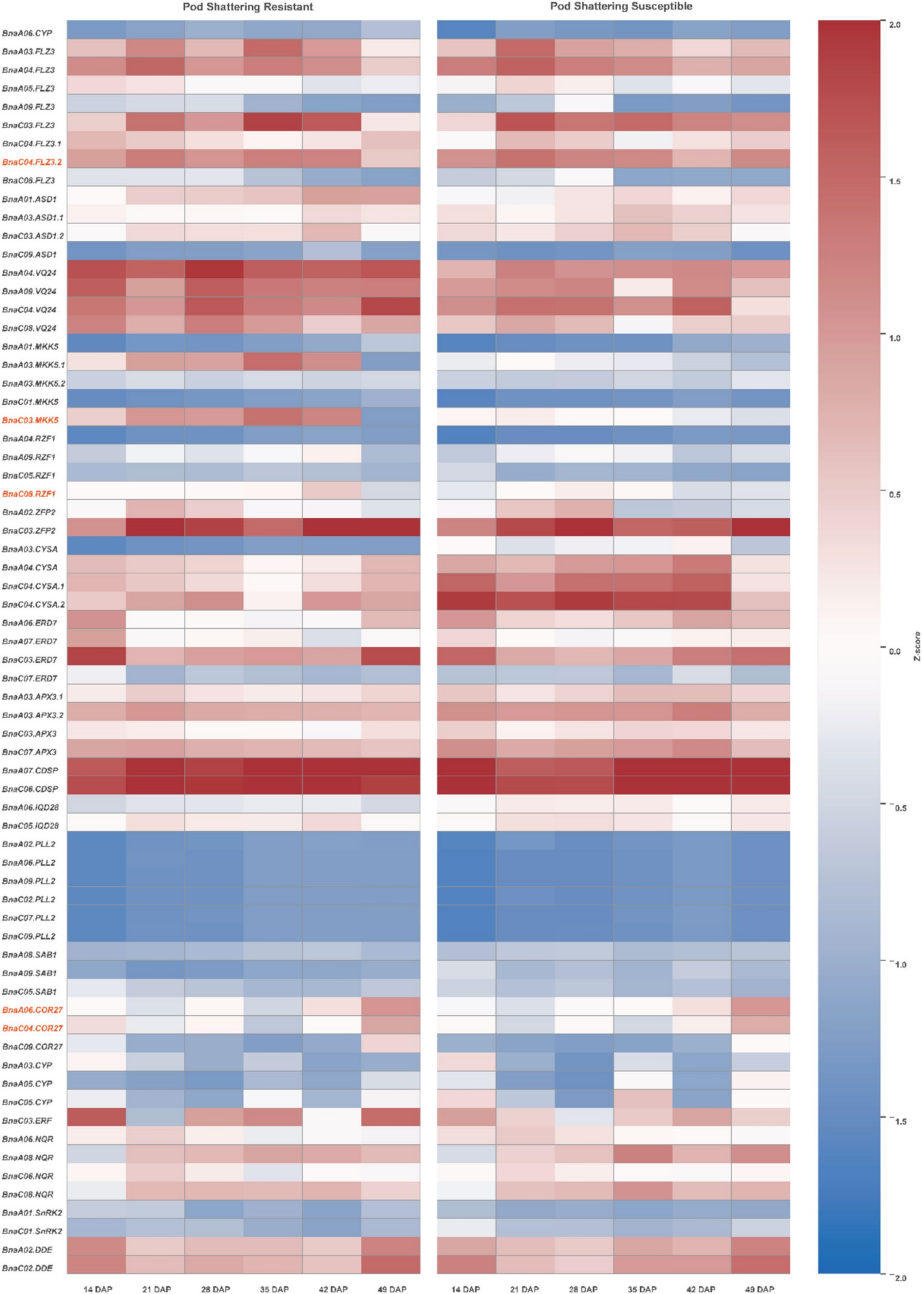
Heatmap of expression profiles of candidate genes associated with pod shattering in *B. napus*. Gene expression patterns are shown for pod-shattering-resistant and susceptible genotypes across six pod developmental stages (14, 21, 28, 35, 42, and 49 days after pollination [DAP]). The heatmap is color-coded based on Z-score normalization, with red indicating higher expression and blue indicating lower expression. Candidate genes included those involved in hormonal signaling, stress response, cell wall modification, starch metabolism, and pod dehiscence. The highlighted gene families (*FLZ3*, *RZF1*, *MKK5*, and *COR27*) exhibited contrasting expression profiles between resistant and susceptible lines

Four candidate genes, *FLZ3* (*Chr1*), *RZF1*, *MKK5* (*Chr2*), and *COR27* (*Chr8*), were co-localized with significant GWAS SNPs, indicating a potential genetic basis for shattering in sesame (Fig. 7a). Expression profiling in *B. napus* revealed that *FLZ3* expression remained stable across most stages but declined sharply at 49 days after planting (DAP) in the resistant line, suggesting a late-stage role in pod shattering (Fig. 8; Table S3). *RZF1* exhibited an earlier decline in the susceptible line than in the resistant line, possibly contributing to shattering susceptibility. *MKK5* exhibited high early- and mid-stage expression in the resistant line but remained consistently low in the susceptible line, indicating its role in reinforcing pod integrity in the resistant line. *COR27* levels increased at later developmental stages in both genotypes, indicating its potential role in regulating pod opening via stress- and hormone-mediated pathways.

## Discussion

This study employed a multi-model GWAS framework to dissect the genetic architecture of three key capsule-shattering traits in sesame: ST, TCB, and BS. Integrating BLINK, FarmCPU, and MLMM models enabled the identification of robust and reproducible MTAs across three consecutive seasons of field-based phenotyping in 200 diverse genotypes. MTAs were detected on chromosomes 1 and 2, while a previously reported region on chromosome 8 was further validated. These genomic regions represent valuable targets for MAS and genomics-assisted breeding aimed at improving shattering resistance in sesame. The combination of association mapping, functional annotation, and cross-species candidate gene analysis provided evidence for biological pathways central to capsule dehiscence, including pod development, cell wall remodeling, and seed dispersal mechanisms. The identification of conserved candidate genes, particularly those orthologous to shattering-related genes *in B. napus*, demonstrates the functional relevance in pod shattering. As functional analysis relies on cross-species expression data, these findings provide only putative evidence for sesame, and experimental validation is still needed.

From a phenotypic perspective, ST emerged as the most relevant trait because of its direct impact on seed viability and harvestability, whereas TCB and BS served as complementary indicators of capsule stability. Although ST was assessed categorically, the underlying variation is likely continuous. Future studies should therefore incorporate objective phenotyping approaches, such as high-resolution image analysis of capsule opening and dehiscence angle, digital caliper, or force-sensor-based measurements of capsule break strength, and time-resolved assessments of shattering under controlled drying or mechanical stress conditions, to capture this finer-scale variation more accurately. ST is governed by MTAs that influence the extent and timing of capsule opening, making it a primary target for breeding (Raman et al. 2014). The TCB reflects beak morphology, which contributes to seed retention under stress factors such as wind and rainfall (Shtein et al. 2016). Although comparatively stable, BS contributes to capsule stability by maintaining a carpel structure (Yol et al. 2021).

Multi-season phenotyping revealed a consistent increase in complete capsule shattering and a significant G × E interaction for ST, underscoring the trait's sensitivity to environmental variation. These findings are consistent with those of earlier studies that described shattering as an environmentally responsive trait (Teboul et al. 2022; Zhang et al. 2018). The broad-sense heritability of ST (*H*^2^ = 0.39) indicates contributions from both genetic and environmental factors contribute to its variation. This intermediate heritability highlights the breeding challenge of capturing genetic variance while managing strong environmental effects. Comparable heritability estimates for pod- or seed-shattering traits have been reported in other crops, demonstrating the polygenic and environmentally dependent nature of these traits. For example, pod shattering in soybean exhibits broad-sense heritability between 0.28 to 0.46, depending on the environmental conditions and population structure (Funatsuki et al. 2014; Tukamuhabwa et al. 2002). In *B. napus*, estimates of siliqua shattering heritability range from 0.32 to 0.48, while in rice, panicle shattering exhibits moderate heritability values ranging from 0.35 to 0.55 (Lee et al. 2016; Wang et al. 2016), indicating that shattering-related traits in diverse crop species are under substantial genetic control. These moderate heritability estimates suggest that shattering can be effectively improved through genetic mapping and selection, supporting the relevance of the MTAs identified for ST in sesame and their potential utility in breeding programs.

BS remained stable across three years, whereas TCB showed moderate year-to-year variation, indicating trait-specific responses to the environmental conditions. These patterns have direct breeding implications, as ST may require evaluation across multiple environments to identify stable genotypes, whereas TCB can be improved through more targeted, environment-specific selection strategies. However, in regions with lower environmental stress, the targeted selection of locally adapted alleles may enhance yield potential without compromising harvestability. Therefore, multi-environment trials are essential for determining whether genotypes maintain broad stability across diverse conditions or show specific adaptations to high-risk environments.

Multivariate PCA and MCA confirmed that phenotypic variation was primarily driven by genetic differences among genotypes, validating the dataset for association mapping. ST was the only trait that exhibited a significant year effect, further supporting its inclusion in environment-focused breeding programs (Gedifew 2024). PCA of the genotypic data revealed a partial population structure with three overlapping clusters (Pop1, Pop2, and Pop3), which is consistent with previous findings that sesame exhibits moderate population stratification shaped by historical selection and gene flow (Wei et al. 2016). A distinct cluster on the PC1 axis indicates genetic divergence and the potential presence of promising alleles. Kinship analysis confirmed these findings, showing subclusters of closely related lines. These results highlight the importance of correcting for kinship and population structure in GWAS to minimize false associations in the results.

LD decay analysis indicated that *r*^*2*^ dropped below 0.2 at ~ 218 kb, representing a moderately fast decay rate suitable for high-resolution mapping. This was faster than the Ethiopian collection, where decay occurred at ~ 1.64 Mb (Tesfaye et al. 2022), suggesting stronger historical recombination and a greater mapping resolution in the Sudanese panel. Compared with the USDA sesame collection (160.69 kb) (Seay et al. 2024), LD decay was slightly longer, reflecting a narrower genetic base in the Sudanese germplasm due to historical selection. Despite the modest SNP count, the 3,636 SNPs provided adequate coverage, as the estimated requirement for genome coverage (~ 1,600 markers for a 350 Mbp genome at this decay rate) was exceeded. This confirms that the marker density in this study was sufficient to capture genome-wide variation at a high resolution.

The multi-model GWAS approach enhanced the MTA resolution and confidence. BLINK provided strong statistical power, FarmCPU effectively reduced false positives, and MLMM accounted for polygenic background effects (Huang et al. 2019; Segura et al. 2012). The convergence of results across models increases confidence in the robustness of these associations. Two SNPs (*Chr2_15649330* and *Chr8_31466064*) were associated with ST, and three SNPs (*Chr1_19419575*, *Chr8_19392181*, and *Chr8_30292484*) were linked to TCB. ST was treated as a binary trait because of the absence of fully indehiscent types, but the observed phenotypic variation indicates an underlying quantitative or threshold trait (Zhang et al. 2024). These findings underline the importance of employing complementary GWAS models to enhance the detection power of complex traits.

The newly identified locus *Chr8_31466064* was positioned at 31.47 Mb, whereas the previously reported shattering-associated marker *S8_5062843* (Yol et al. 2021) was positioned at 5.06 Mb. This ~ 26.41 Mb separation far exceeded the LD decay threshold (218 kb), confirming *Chr8_31466064* as a distinct locus. This distinction also separates it from nearby candidate genes, such as *SiKAN1* and *SiHEC3* (Ju et al. 2024; Teboul et al. 2022; Yol et al. 2021), although further fine-mapping will be required to resolve linkage relationships within this region. Similarly, *Chr2_15649330* represents a candidate locus for ST, contributing to a substantial proportion of phenotypic variance (*R*^2^ = 25%). For TCB, markers *Chr8_19392181* and *Chr8_30292484* were located within a previously reported QTL-rich region, demonstrating the importance of chromosome 8 in regulating capsule traits. *Chr1_19419575* was identified as a previously unreported locus, broadening the genomic landscape underlying beak morphology in sesame.

Functional gene analysis in *B. napus* identified four candidate genes, *FLZ3*, *RZF1*, *MKK5*, and *COR27*, which are involved in cell wall modification, hormone signaling, and stress response regulation. *FLZ3*, a negative regulator of *SnRK1* signaling, modulates ABA-mediated stress responses, and its reduced expression in resistant lines may help maintain pod stability (Xiao et al. 2024). *RZF1*, which encodes a RING-type E3 ubiquitin ligase, regulates protein turnover under stress, and its early downregulation in susceptible lines may weaken stress resilience (Afridi et al. 2022; Han et al. 2022). *MKK5* integrates ABA signaling and developmental cues, with high expression in resistant shattering lines, indicating its role in maintaining pod wall strength (De Zélicourt et al. 2016; Gaur et al. 2018). *COR27*, responsive to ABA and circadian signaling, may coordinate pod opening during late-stage senescence (Jaradat et al. 2014; Panigrahy et al. 2022). Mahmood et al. (2023) highlighted hormone signaling and polygalacturonase activity as key pathways in pod dehiscence, suggesting cross-species regulatory conservation. The identification of sesame orthologs of *FLZ3*, *RZF1*, *MKK5*, and *COR27* supports this hypothesis and demonstrates the potential of comparative genomics for accelerating trait improvement. However, *B*. *napus* expression data provide only putative evidence for sesame. Functional validation in sesame via targeted expression studies, genome editing, or mutant analysis remains essential due to species-specific differences in genome organization, regulatory networks, and developmental timing.

From a breeding perspective, the identified SNPs present direct opportunities for the application of MAS. Thus, practical implementation involves the following steps: (1) validation of the SNPs in independent and diverse germplasm panels to confirm their stability, (2) development of cost-effective and high-throughput genotyping assays, such as KASP markers, (3) integration of validated markers into breeding pipelines for early generation selection, and (4) combining MAS with recurrent selection or genomic selection to capture better the polygenic nature of capsule shattering. Effective deployment also depends on breeder access to genotyping platforms, the establishment of suitable training populations, and integration with regional breeding priorities.

## Conclusions

This study employed multi-model GWAS, multi-season field-based phenotyping, and cross-species candidate gene analysis to identify potential MTAs and candidate genes regulating capsule shattering in sesame. New associations were identified on chromosomes 1, 2, and 8, with *Chr2_15649330* and *Chr1_19419575* representing previously unreported MTAs. The allelic effects of *Chr8_31466064* further demonstrated its role as an independent QTL within the chromosome 8 shattering domain. In silico analysis identified four conserved candidate genes (*FLZ3*, *RZF1*, *MKK5*, and *COR27*) involved in cell wall modification, hormone signaling, and stress response. Together, these results provide high-confidence MTAs and candidate genes that can serve as molecular resources for MAS and genomics-assisted breeding in sesame. Although the study was limited by the single-site evaluation and the absence of fully indehiscent genotypes, the MTAs identified in partially shattering types remain highly relevant. Future efforts should emphasize multi-location trials, functional validation of candidate genes, and a combination of genomic prediction and genome editing to accelerate the development of shattering-resistant sesame cultivars.

## Declaration

## Conflict of interest

The authors declare that they have no conflict of interest.

## Supplementary Information

Below is the link to the electronic supplementary material.

Supplementary file1 (XLSX 35 kb)

## Data Availability

Sequenced reads for the sesame lines used in this study are available under NCBI Bioproject PRJNA1184775. The datasets supporting the conclusions of this study are included within the article and its additional files.
